# Emergence of polymyxin B-heteroresistant hypervirulent *Klebsiella pneumoniae* from an individual in the community with asymptomatic bacteriuria

**DOI:** 10.1186/s12866-022-02462-9

**Published:** 2022-02-07

**Authors:** Jun Li, Mengli Tang, Fengjun Xia, Changhang Min, Yongmei Hu, Haichen Wang, Mingxiang Zou

**Affiliations:** 1 Department of Clinical Laboratory, Xiangya Hospital, Central South University, Changsha, 410008 Hunan China; 2 National Clinical Research Center for Geriatric Disorders, Xiangya Hospital, Central South University, Changsha, 410008 Hunan China

**Keywords:** Hypervirulent *K. pneumoniae*, Polymyxin B heteroresistance, Asymptomatic bacteriuria, Community-dwelling adult, Urine specimen, Whole genome sequencing

## Abstract

**Background:**

The heteroresistance of polymyxin B, a last-resort antibiotic used to treat many serious bacterial infections, may lead to antibiotic treatment failure. However, polymyxin B-heteroresistant isolates are rare in individuals living in the community. We report a polymyxin B-heteroresistant hypervirulent *Klebsiella pneumoniae* (hvKP) isolate from an individual in the community with asymptomatic bacteriuria.

**Results:**

The NYTJ35 isolate had multiple virulence genes that encoded a mucoid phenotype regulator (*rmpA*), aerobactin (*iucABCD*-*iutA*), salmochelin (*iroBCDN*), yersiniabactin (*irp1–2* and *ybtAEPQSTUX*), and a truncated *rmpA2*. Infection of *galleria mellonella* larvae indicated the isolate was hypervirulent. Antimicrobial susceptibility testing showed it was susceptible to all tested antibiotics except polymyxin B. The proportion of surviving bacteria was 1.2 × 10^− 7^ based on the population analysis profile (PAP) method, suggesting the presence of polymyxin B heteroresistance. The isolate was not hypermucoviscous, but it was a strong biofilm producer. It had capsular serotype K1 and belonged to sequence type 23 (ST23). The isolate also had the D150G substitution in *phoQ*, which is known to confer polymyxin B resistance.

**Conclusions:**

We identified the co-occurrence of hypervirulence and polymyxin B heteroresistance in a *K. pneumoniae* isolate from an individual with asymptomatic bacteriuria. We suggest the use of increased screening for hvKP in individuals living in the community.

## Background

*Klebsiella pneumoniae* (KP) is one of the six ESKAPE pathogens (*Enterococcus faecium*, *Staphylococcus aureus*, *Klebsiella pneumoniae*, *Acinetobacter baumannii*, *Pseudomonas aeruginosa*, and *Enterobacter* spp.) that are leading causes of nosocomial infections throughout the world [1]. Hypervirulent *K. pneumoniae* (hvKP) has increased virulence and transmissibility, and is associated with a higher mortality rate [2]. The first report of hvKP was in Taiwan during 1986 [3], and subsequent studies identified hvKP in many other regions, including France, mainland China, Japan, Germany, the United States, Brazil, and Mexico [4–11]. This variant can infect the liver, lungs, and urinary tract. Hosoda et al. recently described a patient from Japan who had COVID-19 and a respiratory infection by hvKP and subsequently died from respiratory failure [7]. Therefore, hvKP is a pathogen that poses a great threat to human health [12].

Earlier studies reported that hvKP was susceptible to all antimicrobials with the exception of ampicillin, to which *K. pneumoniae* has intrinsic resistance. However, more recent studies reported the emergence of drug-resistant hvKP strains, such as those that produce extended spectrum beta-lactamase (ESBL) and others that have carbapenem resistance [4–6]. Until recently, polymyxin B was regarded as the last-resort antibiotic for the treatment of serious infections by multidrug resistant (MDR) Gram-negative bacteria. Unfortunately, several recent reports described nosocomial infections by polymyxin B-resistant hvKP [13, 14]. The presence of polymyxin B resistance is a significant challenge due to the limited availability of alterative effective antimicrobials. The co-occurrence of hypervirulence and MDR in *K. pneumoniae* is therefore a significant clinical challenge.

Heterogeneous antibiotic resistance may be considered a stage in the progression to antibiotic resistance [15]. There is currently only limited knowledge of polymyxin B-heteroresistance in hvKP, and the specific molecular and epidemiological characteristics of these infections are still unknown [16]. Asymptomatic bacteriuria (ASB), an common type of urinary tract infection (UTI), is defined by a positive urine culture without signs and symptoms in the patient. ASB mainly occurs in community-dwelling individuals infection [17]. ASB is more in the elderly, in that the prevalence is less than 2% for children and up to 50% in elderly residents of long-term care facilities [17]. The present study describes the isolation of a polymyxin B-heteroresistant hvKP strain from the urine sample of an asymptomatic male individual living in the community, and the molecular epidemiological characteristics of this strain, as a basis for preventing transmission.

## Results

### Characteristics and identification of the hvKP isolate

During routine mass screening for kidney disease, we found a *K. pneumoniae* isolate (NYTJ35) in the urine sample of an asymptomatic male individual who was 34 years-old and living in the community. This individual had no symptoms of UTI, and therefore received no treatment. However, his urine tested positive for white blood cells, protein, and nitrite. After collection and culturing of the uncontaminated urine, the colony count was 1 × 10^5^ colony forming units (CFU)/mL.

### Antimicrobial susceptibility and gene resistance testing

The antimicrobial susceptibility tests (Table 1) showed that NYTJ35 was sensitive to all tested antibiotics, including ceftazidime (CAZ), cefepime (FEP), imipenem (IPM), meropenem (MEM), ceftazidime/avibactam (CZA), aztreonam (ATM), piperacillin/tazobactam (TZP), nitrofurantoin (NIT), amikacin (AMK), levofloxacin (LVX), and tigecycline (TGC), but not polymyxin B (2 μg/mL). We used the population analysis profile (PAP) method, the gold standard for detection of heteroresistance, to detect polymyxin B heteroresistance in NYTJ35. Thus, when the polymyxin B concentration was 8 μg/mL (4-fold above its breakpoint of 2 μg/mL), the number of colonies in the stock solution was 37 CFU; a control without polymyxin B led to 301 CFU at a dilution of 10^− 6^. As described in the Methods, this indicated that the proportion of surviving bacteria was 1.2 × 10^− 7^ (between 10^− 7^ and 50%) and that this strain was heteroresistant to polymyxin B.

**Table 1:** Minimum inhibitory concentrations of different antibiotics for the NYTJ35 isolate^a^

Antibiotic	MIC (μg/mL)
Ceftazidime	≤1
Cefepime	≤1
Imipenem	≤0.5
Meropenem	≤0.25
Ceftazidime/avibactam	≤1
Aztreonam	≤1
Piperacillin/tazobactam	16
Nitrofurantoin	≤32
Amikacin	≤4
Levofloxacin	0.25
Tigecycline	1

^a^Determined using broth microdilution

The NYTJ35 strain had the D150G substitution in *phoQ*, but had no mutations in the chromosomal genes *mgrB*, *phoP*, *pmrA*, or *pmrB*. We also did not detect the plasmid mediated *mcr*-like gene, suggesting this mutation may be responsible for the heteroresistance to polymyxin B.

### Virulence factors

When grown on agar plates, the colony morphology of NYTJ35 was not mucoid and only formed strings less than 5 mm, indicating it was not hypermucoviscous. The strain had a K1 capsule and multiple virulence genes encoding a mucoid phenotype regulator (*rmpA*), aerobactin (*iucABCD*-*iutA*), salmochelin (*iroBCDN*), and yersiniabactin (*irp1–2* and *ybtAEPQSTUX*). Notably, the isolate carried a truncated *rmpA2* gene.

### Biofilm formation

Crystal violet staining indicated that NYTJ35 was a strong biofilm producer. (OD > 4 × ODc).

### *Galleriamellonella* infection model

**Fig. 1:**
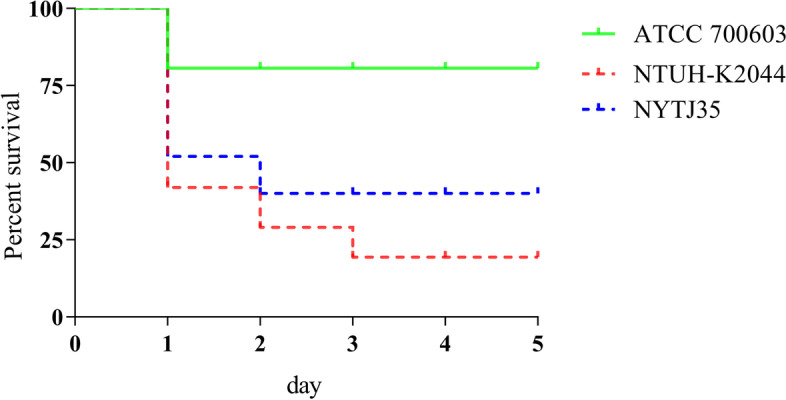
Virulence of the NYTJ35 isolate. The *G. mellonella* infection model was used to determine the virulence of ATCC 700603 (low-virulence KP control), NTUH-K2044 (high-virulence KP control), and the NYTJ35 isolate

Analysis of the virulence of NYTJ35 indicated that the survival of *G. mellonella* larvae was 40.0%, higher than from infection by ATCC 700603 and lower than from infection by NTUH-K2044. These results thus indicated that this strain was hypervirulent (Fig. 1).

### Phylogenic analysis

**Fig. 2:**
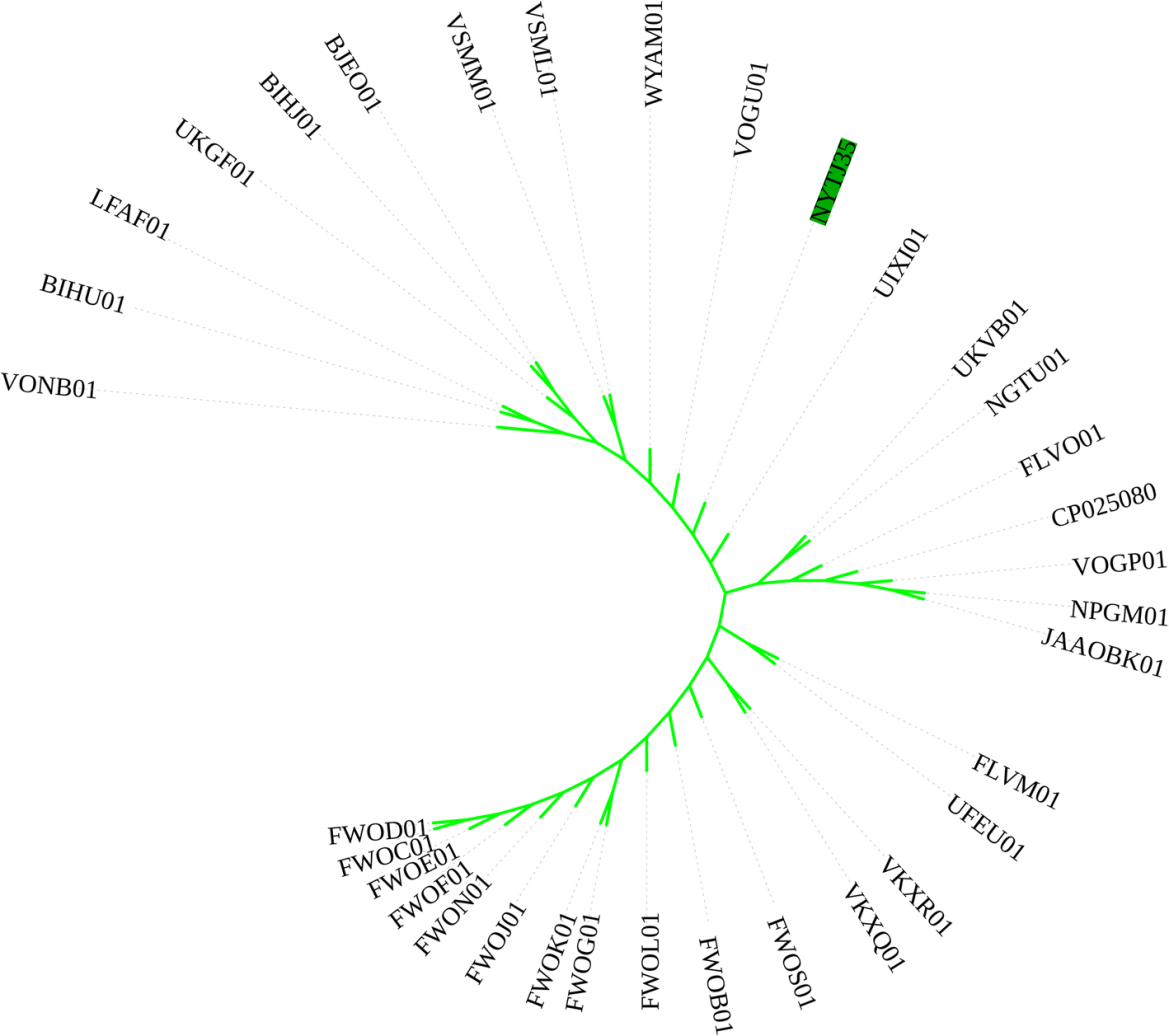
Phylogenetic analysis of NYTJ35. Phylogenic analysis was performed using BacWGSTdb 2.0 with a single nucleotide polymorphism (SNP) threshold of 500, the MLST scheme with cgMLST, and a MLST threshold of 200. The cgMLST strategy led to the identification of 33 isolates that were in two STs (ST23: *n* = 32; ST57: *n* = 1). NYTJ35 was most closely related to ST23 FLVM01 (GenBank: FLVM00000000.1), with 108 different alleles

We deposited the sequence data in the NCBI (PRJNA753708). The multi-locus sequence typing (MLST) result showed it belonged to ST23 (2-1-1-1-9-4-12). We performed phylogenetic analysis of NYTJ35 using BacWGSTdb 2.0 (SNP threshold: 500, MLST scheme with cgMLST, MLST threshold: 200). Based on the cgMLST strategy, we identified 33 close isolates, with 32 isolates in ST23 and 1 isolate in ST57. The most closely related isolate was ST23 FLVM01 (GenBank: FLVM00000000.1), with 108 different alleles. FLVM01 was from a human urine sample collected in Thailand on 13 June 2016 (Fig. 2).

## Discussion

hvKP is highly pathogenic and transmissible, and the control of hvKP infections is challenging due to the limited availability of effective anti-infective therapies. Moreover, there is increasing drug resistance in hvKP. In particular, ESBL-producing strains have emerged, there is evidence of carbapenem resistance in nosocomial hvKP infections, and polymyxin B-resistant hvKP was recently identified. In particular, Gu et al. [18] detected polymyxin B-resistant hvKP in an infant with diarrhea and Huang et al. [19] reported polymyxin B-resistant hvKP in clinical specimens. Polymyxin B-heteroresistance may be considered a stage during the progression to antibiotic resistance, and patients with these infections usually experience treatment failure. Previous studies also detected polymyxin B-heteroresistant isolates in classical *K. pneumoniae* (cKP) [20, 21], but few studies examined polymyxin B-heteroresistance in hvKP [16], especially from individuals in the community. Thus, the prevalence of polymyxin B-heteroresistance in hvKP in the community may be underestimated [22]. We isolated a polymyxin B-heteroresistant hvKP strain (NYTJ35) from the urine sample of a male individual in the community who had ASB, and analyzed its molecular and epidemiological characteristics to provide a basis for preventing transmission.

There is currently no agreement on the definition of hvKP. Initially, hypermucoviscosity (defined by a positive string test) was considered a critical characteristic of hvKP [23]. However, some studies found that certain hvKP strains did not have the hypermucoviscous phenotype [24]. Recent studies showed that multiple biomarkers that were common on the hvKP virulence plasmid, such as _*p*_*rmpA*, _*p*_*rmpA2*, *iucA*, *iroB*, and *peg-344*, provided highly accurate identification of hvKP [25, 26]. The present study examined virulence-related genes, hypermucoviscosity, capsular serotype, and virulence of the strain using the *G. mellonella* larva test.

We found that NYTJ35 was heteroresistant to polymyxin B, but was susceptible to other antimicrobial agents, in accordance with a report by Lu et al. [13] that described hypervirulent *K. variicola*. The most common mechanism of polymyxin B resistance is chromosomal mutation in genes such as *pmrA*, *pmrB*, *phoP*, *phoQ*, and *mgrB*. The D150G substitution in *phoQ*, one of the most common chromosomal mutations responsible for polymyxin B resistance [14], was also present in NYTJ35. Cheong et al. reported colistin heteroresistance in *K. pneumoniae* isolates due to diverse mutations of *PmrAB* and *PhoPQ* in resistant subpopulations, although they did not detect other chromosomal gene mutations in their isolates [27]. The *mcr* plasmid gene is a transferable polymyxin B resistance gene first discovered in 2015. Although this gene confers drug resistance transfer between humans and animals [28], it was not present in NYTJ35.

The hypermucoviscosity phenotype is an important feature of hvKP, but not all hvKP isolates have hypermucoviscosity [24]. NYTJ35 has genes encoding the mucoid phenotype regulator (*rmpA*), but had a truncated *rmpA2* that presumably led to the non-hypermucoviscous phenotype, suggesting that the loss of hypermucoviscosity in polymyxin B-heteroresistant hvKP may increase its fitness [29]. In particular, the loss of hypermucoviscosity may reduce adhesion of these bacteria to human cells, such as neutrophils [30]. Notably, although NYTJ35 was not hypermucoviscous, it was a strong biofilm producer. This may be related to its ability to escape immune cells in the urinary tract and thus increase colonization [31].

There are multiple serotypes of hvKP, but most isolates are K1 or K2 [32]. Previous studies showed that isolates with the K1 serotype were mainly in ST23, whereas isolates with the K2 serotype were in many STs, including ST25, ST65, ST66, and ST86 [33–36]. We found that NYTJ35 had the K1 serotype and was in ST23, an ST common in hospital-acquired and community-acquired infections. We also found that this isolate’s closest known relative was FLVM01 (GenBank: FLVM00000000.1), although they had 108 different alleles, suggesting they may only be distantly related.

## Conclusion

To our knowledge, this study is the first to detect a polymyxin B-heteroresistant hvKP isolate in a urine specimen of an individual from the community with asymptomatic bacteriuria. The polymyxin B-heteroresistant strain described here possibly had increased fitness due to its loss of hypermucoviscosity, which could impede its adhesion to immune cells. The co-occurrence of hypervirulence and polymyxin B heteroresistance in a *K. pneumoniae* isolate from an asymptomatic individual living in the community suggests the need for increased surveillance.

## Methods

### Bacterial strain

On 20 November 2020, NYTJ35 was isolated at Xiangya Hospital of Central South University (Changsha, China) from the urine sample of a healthy male individual who was living in the community. This isolate was identified using matrix-assisted laser desorption/ionization time-of-flight mass spectrometry (MALDI-TOF MS; Bruker Daltonics GmbH, Bremen, Germany). Briefly, one colony from an overnight culture was taken with a disposable loop and spotted onto a metal plate and the spots were then covered with 1 μL of a α-cyano-4-hydroxy-cinnamic acid (HCCA) matrix (Bruker Daltonik GmbH, Bremen, Germany). Then, bacterial samples on the microplate were analyzed with MALDI-TOF MS. Finally, MALDI Biotyper® (Bruker Daltonik GmbH, Bremen, Germany) software was used to classify the isolate at the genus and species level. The quality control strain was *Escherichia coli* ATCC 25922 (National Center for Clinical Laboratories, Beijing, China).

### Antimicrobial susceptibility testing

The broth microdilution method was used to determine the minimum inhibitory concentrations (MICs) of the following antimicrobial agents: CAZ, FEP, IPM, MEM, CZA, ATM, TZP, NIT, AMK, LVX, TGC, and polymyxin B. All results were interpreted according to Clinical and Laboratory Standards Institute (CLSI) [37]. The MIC of TGC was reported in accordance with the breakpoint established by the U.S. Food and Drug Administration. *E. coli* ATCC 25922 was used for quality control.

Polymyxin B-heteroresistance in hvKP was determined by the PAP method, the gold standard for detecting heteroresistance [38]. Briefly, this technique quantifies the proportion of resistant cells within a culture in response to different antibiotic concentrations. Heteroresistance was considered present when the proportion of surviving bacteria at a polymixin B concentration at least 4-fold above its breakpoint was between 10^− 7^ and 50%. This was calculated as: (number of colonies on polymyxin B plate × dilution factor)/(number of colonies on antibiotic free plate × dilution factor).

### Detection of capsular serotypes, mucoviscous phenotype, and biofilm formation

After overnight culturing, the genomic DNA of NYTJ35 was extracted using a boiling method [39]. The polymerase chain reaction (PCR) was used to detect capsular serotype genes (K1, K2, K5, K20, K54, K57) using primers as previously described [2]. Positive PCR products were then subjected to direct Sanger sequencing.

The mucoviscous phenotype was evaluated using the string test [40]. The isolate was cultured overnight on a blood agar plate at 37 °C, and a bacterial colony was then stretched with an inoculation loop. Hypermucoviscosity was defined by the formation of viscous strings that were at least 5 mm in length.

Crystal violet staining was used to test biofilm formation by NYTJ35 [41]. Absorbance was measured at 570 nm, and data are presented as the means ± standard deviations of assays performed in triplicate. *K. pneumoniae* ATCC 700606 and NTUH-K2044 were used as negative and positive controls, respectively. The optical density cut-off (ODc) value was determined using a previously described formula [42]: ODc = average OD of the negative control + (3 × standard deviation of the negative control). Then, the ability of the isolate to produce biofilm was categorized as: strong (OD > 4 × ODc); medium (4 × ODc ≥ OD > 2 × ODc); weak (2 × ODc ≥ OD > ODc); or none (OD ≤ ODc).

### *Galleria mellonella* infection model

The virulence of NYTJ35 was evaluated using the *G. mellonella* larvae infection model (Tianjin Huiyude Biotech Company, Tianjin, China) [2]. An overnight culture of *K. pneumoniae* was adjusted to 1 × 10^8^ CFU/mL using phosphate-buffered saline. Then, *G. mellonella* larvae were injected with 10 μL of the culture and incubated in the dark at 37 °C for 5 days, with continuous monitoring of survival. *K. pneumoniae* NTUH-K2044 and *K. pneumoniae* ATCC 700603 were used as high-virulence and low-virulence controls, respectively. All experiments were performed in triplicate.

### Whole-genome sequencing (WGS)

WGS was used to identify resistance genes and virulence factors in the hvKP isolate. Approximately 10 μg of DNA was extracted using the DNeasy UltraClean Microbial Kit (QIAGEN, Hilden, Germany) to establish two Illumina paired-end libraries with 500 and 2000 base pairs (average insertion lengths). Reads with any of the following characteristics were excluded from the raw data: (*i*) undefined bases of 5 bp, (*ii*) low-quality (≤ Q20) bases of 20 bp, (*iii*) contamination of adapter, or (*iv*) duplicates. SOAPdenovo version 1.05 was used to assemble the final cleaned reads, and the genome coverage for each strain was approximately 100×. The CGE server (https://cge.cbs.dtu.dk) was used to identify resistance genes. A phylogenic tree was constructed using the BacWGSTdb server with core genome multilocus sequence typing (cgMLST) [43]. The Oxford scheme was used for multilocus sequence typing (MLST), and the sequence types (STs) were determined using the MLST database (https://bigsdb.pasteur.fr/klebsiella/klebsiella.html).

## Data Availability

The datasets generated and analyzed during the current study are available in the NCBI BioProject repository, BioProject accession number: PRJNA753708.

## Abbreviations

ST: Sequence type
hvKP: Hypervirulent *K. pneumoniae*
KP: *K. pneumoniae*
ESBL: Extended spectrum beta-lactamase
MDR: Multidrug resistant
cKP: Classical *K. pneumoniae*
MALDI-TOF MS: Matrix-assisted laser desorption/ionization time-of-flight mass spectrometry
CFU: Colony forming units
MIC: Minimum inhibitory concentrations
CAZ: Ceftazidime
FEP: Cefepime
IPM: Imipenem
MEM: Meropenem
CZA: Ceftazidime/avibactam
ATM: Aztreonam
TZP: Piperacillin/tazobactam
NIT: Nitrofurantoin
AMK: Amikacin
LVX: Levofloxacin
TGC: Tigecycline
PCR: Polymerase chain reaction
OD: Optical density
cgMLST: Core genome multilocus sequence typing
MLST: Multilocus sequence typing
SNP: Single nucleotide polymorphism
WGS: Whole-genome sequencing
CLSI: Clinical and Laboratory Standards Institute
PAP: Population analysis profile
HCCA: A α-cyano-4-hydroxy-cinnamic acid
ASB: Asymptomatic bacteriuria
UTI: Urinary tract infection
